# mTORC2 inhibition reduces tumor burden via STAT1 activation and enhanced response to anti–PD-L1 therapy

**DOI:** 10.1038/s41419-025-08367-5

**Published:** 2025-12-22

**Authors:** Anna Gschwendtner, Birgit Schütz, Madalina A. Mirea, Oliver Eckel, Mikolaj Z. Kepa, Stephanie Deborah Fritsch, Raimund Oberle, Thomas Weichhart, Markus Hengstschläger, Mario Mikula

**Affiliations:** 1 Institute of Medical Genetics, Center for Pathobiochemistry and Genetics, Medical University of Vienna, Vienna, Austria; 2 Institute of Medical Chemistry, Center for Pathobiochemistry and Genetics, Medical University of Vienna, Vienna, Austria

**Keywords:** Melanoma, Immunosurveillance

## Abstract

Although melanoma treatment has progressed considerably in recent years, increasing patient response rates remains a significant challenge. The interferon pathway is known to promote immune recognition, but its sustained activation can contribute to adaptive immune exhaustion. In this study, we demonstrate that myeloid-specific deletion of Rictor in a mouse melanoma model enhances STAT1 signaling while reducing PD-L1 expression. Furthermore, IFN-γ–activated macrophages inhibited melanoma growth in a human skin organoid model. Notably, in vivo inhibition of AKT, in conjunction with anti–PD-L1 therapy, suppressed tumor progression. Mechanistically, we identified IFN-γ–mediated downregulation of IGF-1 as a key event during inflammation, and showed that supplementation with recombinant IGF-1 dampens STAT1 activation. Our findings reveal that targeting the Rictor-AKT axis induces a dual effect - boosting pro-inflammatory signaling while downregulating immunosuppressive factors such as PD-L1 and IGF-1. These results support the potential of AKT inhibitors to enhance the efficacy of immune checkpoint therapies in melanoma patients.

## Introduction

Malignant melanoma has the highest mortality rate among all skin cancer types. Its incidence is increasing worldwide and is predicted to rise further in the coming decades [1]. Particularly in advanced stages, melanoma is challenging to treat, as many patients show therapy resistance and relapse, underlying the need for novel treatment options [2, 3]. Immune checkpoint blockade targeting the PD-1/PD-L1 pathway has shown remarkable success in melanoma [4]. Nonetheless, only a subset of patients benefits, prompting efforts to enhance therapeutic efficacy [5, 6].

Part of the treatment resistance can be attributed to tumor-infiltrating immune cells supporting the tumor, including tumor-associated macrophages (TAMs). TAMs can either show an anti-inflammatory or pro-inflammatory phenotype, which can influence tumor initiation and progression [7–9]. Interestingly these changes can be triggered by modulation of the mTOR pathway. The pathway consists of the two complexes mTORC1, containing the adaptor protein Raptor, and mTORC2, containing the adaptor protein Rictor [10, 11]. Since generation of pro-inflammatory TAMs by myeloid-specific deletion of Rictor caused enhances colorectal cancer tumorigenesis [12], we aimed to apply this model to explore its consequences for melanoma growth and therapeutic treatment. Furthermore, while it is widely recognized that activation of mTORC1 in myeloid cells promotes the development of anti-inflammatory macrophages, and that Rictor deficiency leads to the emergence of pro-inflammatory macrophages, the underlying mechanisms driving this process remain poorly understood [13, 14].

In this study, we show that tumors grow significantly smaller in mice harboring a myeloid-specific Rictor KO. KO tumors displayed high macrophage infiltration, demonstrating an inflammatory phenotype hallmarked by an increase in IFN-γ triggered STAT1 signaling and MHCII expression. Importantly, we found a downregulation of PD-L1 as well as IGF-1. In a novel human organoid model treated with MK-2206 to mimic the loss of Rictor, we were able to show the importance of macrophages for a decrease in melanoma tumor size. Lastly, we were able to show that co-administration of MK-2206 in mice is able to enhance the effect of single PD-L1 treatment.

## Results

### Macrophage-specific Rictor loss reduces melanoma tumor growth

**Fig. 1:**
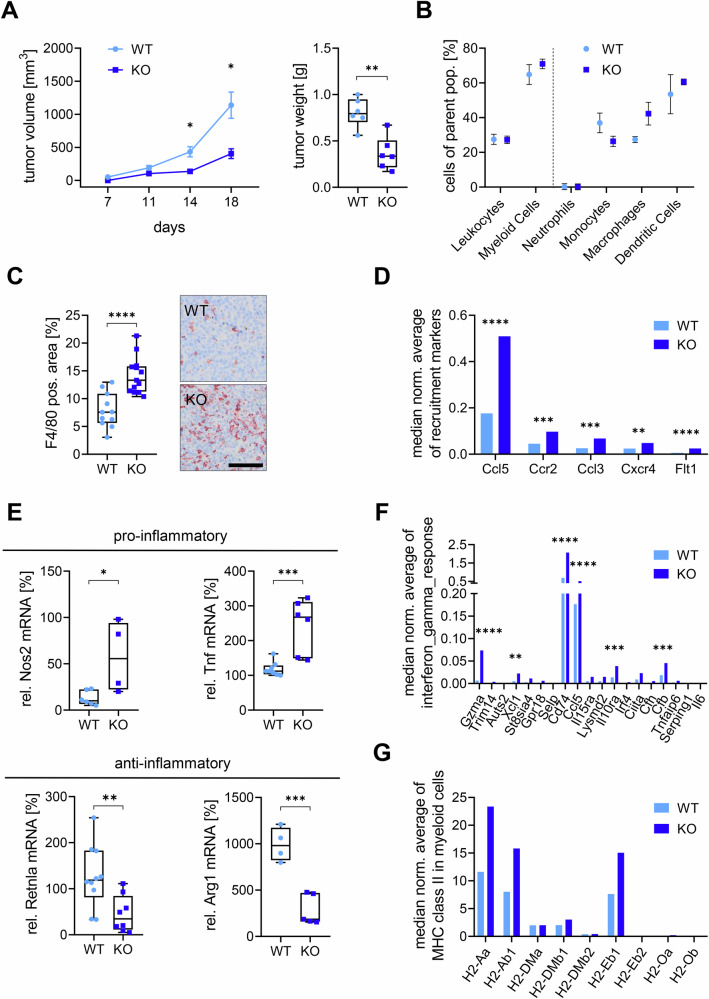
Myeloid-specific Rictor knockout leads to reduced tumor growth and heightened inflammation. **A** B16-F10 melanoma cells were implanted in Rictor WT and KO mice, and tumor growth was monitored until the largest tumors reached over 1000 mm³. Tumor volume (mm³) and weight (g) are presented as mean ± SEM. Statistical analysis was performed using a mixed-effects model and unpaired t test (*N* = 6). **B** Tumor-infiltrating myeloid cells were quantified by flow cytometry using markers CD45, CD11b, Ly6G, Ly6C, F4/80, CD11c, and CD24. Percentages of parent populations are shown as mean ± SEM (*N* = 3). **C** Macrophages in paraffin-embedded tumor sections were stained using the F4/80 marker. AEC-positive area [%] is displayed as individual values in a box-and-whisker plot. Scale bar: 100 µm. Statistical analysis: unpaired t test. Sample sizes: WT = 11, KO = 13. **D** Gene expression analysis of WT and KO tumors shows median normalized expression levels of recruitment markers. **E** Tumor-associated macrophages were sorted and analyzed by qPCR for pro-inflammatory genes (Nos2, Tnf) and anti-inflammatory genes (Retnla, Arg1). Relative mRNA levels [%] are shown as individual values in box-and-whisker plots. Statistical analysis: unpaired t test (*N* = 5). **F**, **G** Gene expression analysis of whole tumors showing median normalized expression of the (**F**) HALLMARK_INTERFERON_GAMMA_RESPONSE gene set and (**G**) MHC class II isoforms in myeloid cells. *P*-values: * ≤ 0.05, ** ≤ 0.01, *** ≤ 0.001, **** ≤ 0.0001.

To gain insight into how mTORC2 in macrophages affects melanoma formation and progression, B16-F10 tumor cells were injected into Rictor WT and KO LysMcre mice. Significantly smaller tumors formed in the KO mice compared to WT mice, as measured by tumor volume and tumor weight (Fig. 1A). Parts of each tumor were digested to obtain single cells, while remaining tissue was used for histologic processing. Analysis of tumor tissue by flow cytometry showed an increase in macrophages, but a decrease in monocytes in KO mice, while other cell populations were not affected (Fig. 1B). The increase in the macrophage numbers was verified through a F4/80 staining of histologic tumor sections (Fig. 1C), while staining of lymphocytes only showed modest upregulation (Fig. S1A). To explore for changes in chemoattractants and immune-related recruitment markers, we generated gene expression data for the whole tissue and visualized selected genes (Fig. 1D). The results indicate that more monocytes are recruited into the tumor, which can further differentiate into macrophages, matching the result in Fig. 1B. As macrophages are highly plastic cells known to polarize between a pro- and anti-inflammatory phenotype [15], we next isolated TAMs by flow cytometric sorting and analyzed markers indicating polarization states. We found a significant increase in the pro-inflammatory markers Nos2 and Tnf as well as a significant decrease in the anti-inflammatory markers Retnla (protein Fizz-1) and Arg1 (Fig. 1E). Additionally, we tested for upregulated gene sets and identified the hallmark gene set for IFN gamma response (Fig. 1F), as well as the IFN-γ target members of the MHCII family (Fig. 1G, Fig. S1B). These results indicate an inflammatory tumor microenvironment in KO mice through changes in both the number as well as the phenotype of macrophages.

### Inflammatory pathways are activated in Rictor KO tumors and MK-2206 treated cells

**Fig. 2:**
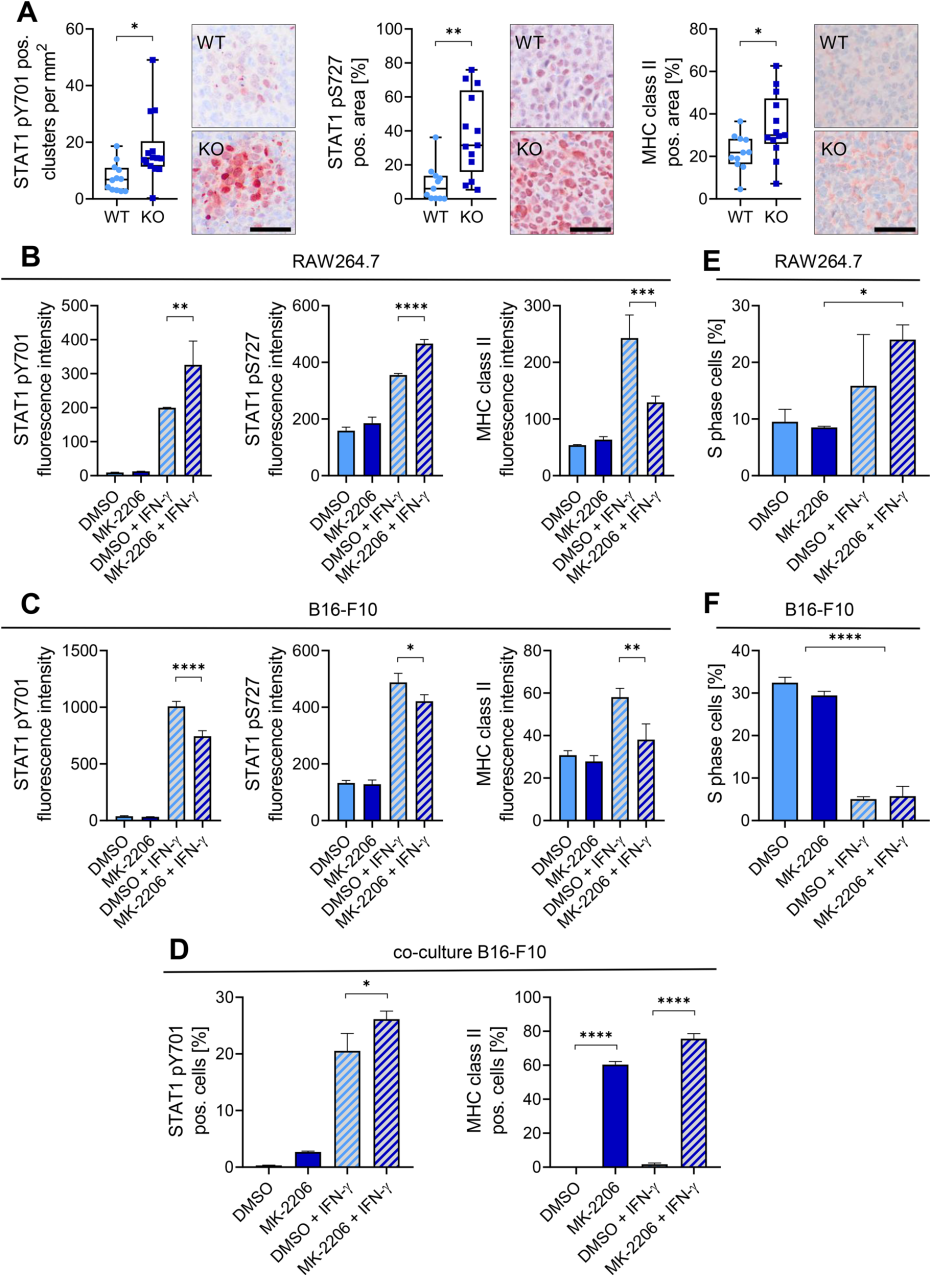
Myeloid-specific Rictor knockout enhances STAT1 activation in tumors, with macrophage-specific responsiveness to MK-2206 treatment in vitro. **A** Paraffin-embedded tumor sections were stained for phosphorylated STAT1 (pY701, pS727) and MHC class II. The number of AEC-positive clusters and percentage of AEC-positive area are presented as individual values in box-and-whisker plots. Statistical analysis was performed using an unpaired t-test. Scale bars: 50 µm. Sample sizes: WT = 11, KO = 13. **B**, **C** STAT1 activation and MHC class II expression were measured by in-cell western assay in (**B**) RAW264.7 and (**C**) B16-F10 cells treated for 24 h in starved medium with DMSO, MK-2206 (10 or 1 µM), DMSO + 50 U/mL IFN-γ, or MK-2206 + IFN-γ. Data are presented as mean fluorescence intensity with SD. Statistical analysis: one-way ANOVA (*N* = 3). **D** RAW264.7 and B16-F10 cells were co-cultured and treated for 24 h in starved medium under the same conditions. STAT1 pY701 and MHC class II expression in B16-F10 cells was assessed by flow cytometry. Cell types were distinguished using surface markers CD146, CD45.2, CD11b, and F4/80. Data are shown as mean with SD. Statistical analysis: one-way ANOVA (*N* = 3). **E**, **F** Cell cycle analysis was performed in (**E**) RAW264.7 and (**F**) B16-F10 cells treated under the same conditions as above. Percentage of cells in S phase was quantified using EdU flow cytometry. Results are shown as mean with SD. Statistical analysis: one-way ANOVA (*N* = 3). *P*-values: * ≤ 0.05, ** ≤ 0.01, *** ≤ 0.001, **** ≤ 0.0001.

Since we observed an increase in pathways associated with inflammatory response, we further investigated tumor protein lysates for the activating phosphorylation sites Tyr701 and Ser727 of STAT1 (Fig. S2A). Importantly, STAT1 activation as well as MHCII was significantly increased in tumor tissue, validating our prior data (Fig. 2A). The pharmacological AKT inhibitor MK-2206 can be used to mimic the Rictor KO, as the main target of mTORC2 is AKT. This inhibitor is widely used in research and also in clinical trials for advanced solid tumors [16, 17]. To see if MK-2206 has a similar effect on the inflammatory pathways, we treated RAW264.7 macrophages with the inhibitor alone or in combination with IFN-γ. We found an increase in both activating phosphorylation sites of STAT1 on macrophages in the combination treatment compared to IFN-γ, while the marker MHCII was decreased, indicating inhibition by MK-2206 (Fig. 2B, Fig. S2B). To see if these treatments also effect melanoma cells, we repeated the experiment with B16-F10 cells and observed a decrease in the activating phosphorylation sites of STAT1 and MHCII (Fig. 2C, Fig. S2C). Interestingly, when we co-cultured these cells and repeated the treatment, an increase in both STAT1 pY701 as well as MHCII in both cell types was seen (Fig. 2D, Fig. S3A, Fig. S4A, B). As the tumor size decreased, while the macrophage content in the tumors increased, we checked the effect of the treatments on cell proliferation. While RAW264.7 cells showed increased proliferation upon the combination treatment, B16-F10 cells proliferation rapidly declined as soon as IFN-γ was added (Fig. 2E, F). These data underline the importance of IFN-γ for the effect on inflammation and show that the pharmacological AKT inhibition has a similar effect to Rictor KO.

### MK-2206 and the presence of macrophages leads to smaller melanoma tumor size and increased inflammation in a human skin organoid model

**Fig. 3:**
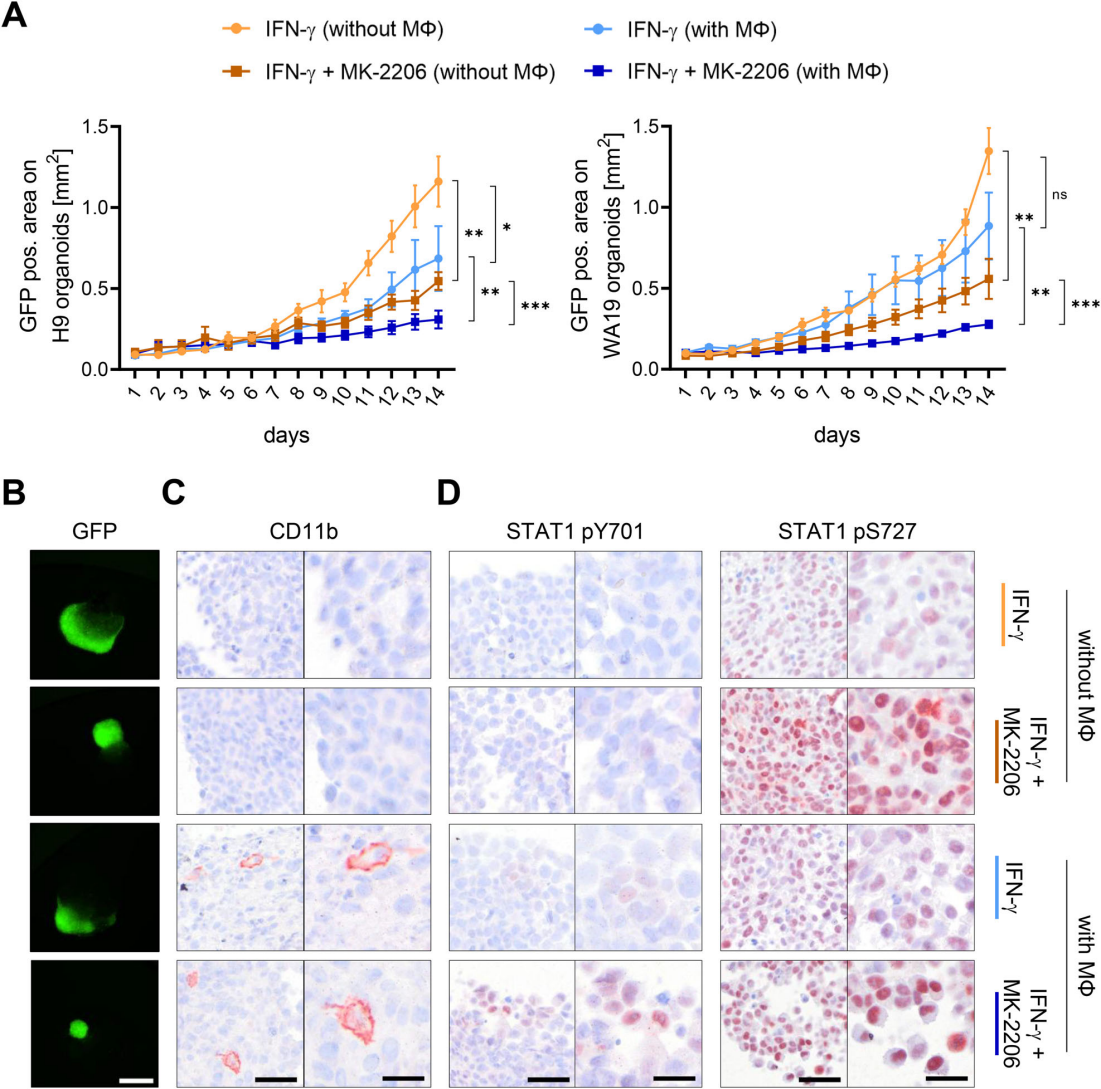
Human skin organoids show smaller tumor growth and increased STAT1 signaling upon MK-2206 treatment and addition of macrophages. **A** Size of GFP-labeled MCM1DLN tumor spheroids on H9 (left panel) or WA19 (right panel) skin organoids over 14 days. Tumor spheroids consisted of just melanoma cells or a mixture of melanoma cells and macrophages. Organoids were treated with either 10 U/mL hIFN-γ or hIFN-γ + 1 µM MK-2206. GFP positive area [µm^2^] is shown as mean ± SEM. Mixed-effects model was used for statistical analysis (*N* = 7). **B** Representative pictures of GFP labeled spheroids on H9 organoids on day 14. Scale bar = 1 mm. **C**, **D** Representative pictures of (**C**) CD11b, (**D**) STAT1 pY701 and STAT1 pS727 stainings of the paraffin-embedded tumor spheroid on organoids. Scale bars: left = 50 µm, right = 25 µm. *P*-values: * ≤ 0.05, ** ≤ 0.01, *** ≤ 0.001.

We next applied an organoid model composed of solely neuro-ectodermal tissue derived from embryonic stem cells, which were grafted with human macrophages and melanoma cells to test the effect on tumor growth. Based on the cell culture results from Fig. 2, again IFN-γ was added to both the control as well as the inhibitor condition. We found a significant decrease in tumor tissue size after the inhibitor treatment compared to the control treatment, being the case for both spheroid types (Fig. 3A, B, Fig. S5A). Moreover, we found that the addition of macrophages alone was enough to decrease the tumor growth (Fig. 3C, Fig. S5B). We next checked the activating phosphorylation sites of STAT1 and observed an increase in signal when MK-2206 was additionally applied to tumor organoids with and without macrophages (Fig. 3D, Fig. S5C). These results highlight the importance of IFN-γ treated macrophages to establish an anti-tumor effect in our organoid system.

### MK-2206 treatment increases the effects of anti-PD-L1 treatments in vivo

**Fig. 4:**
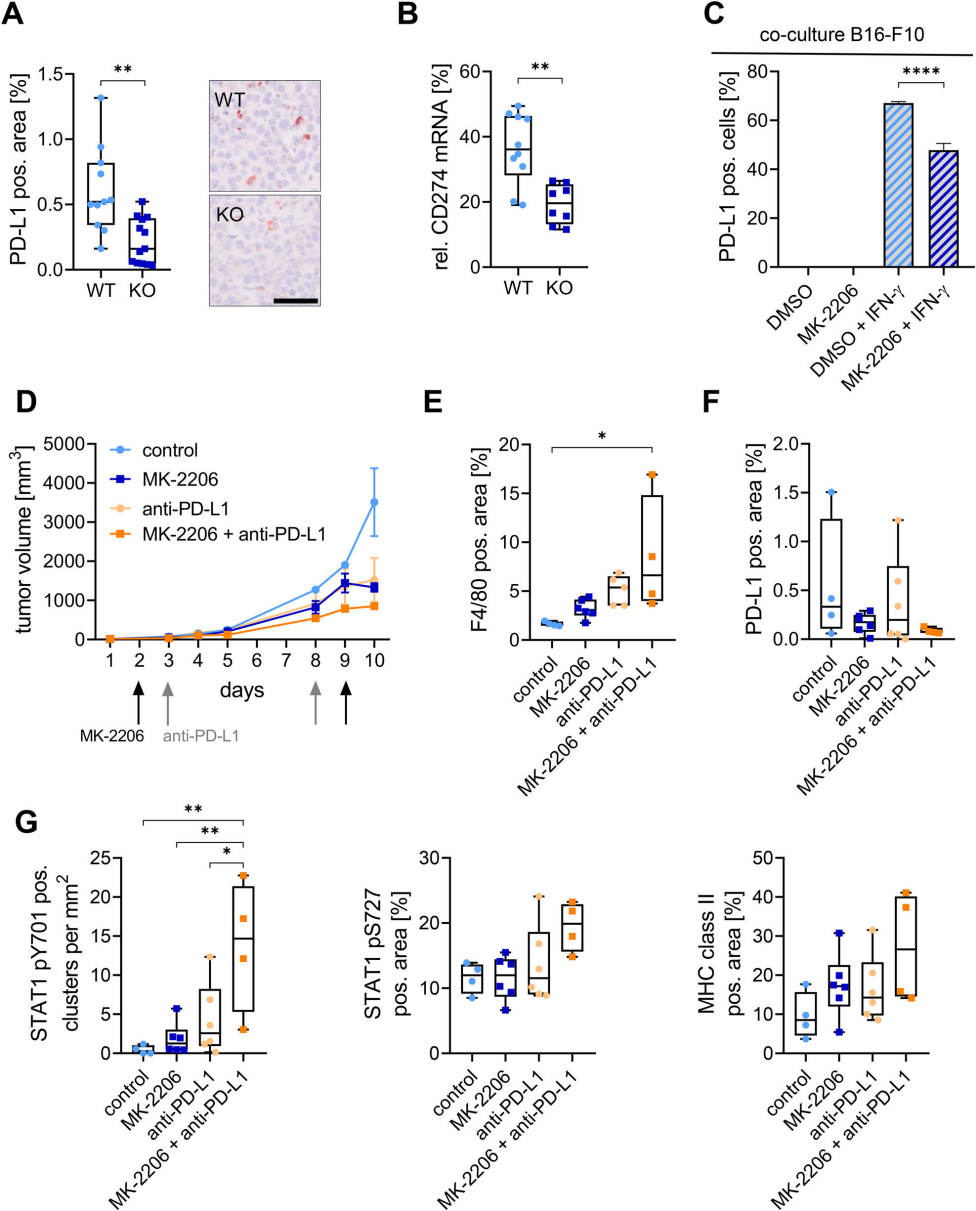
MK-2206 and anti-PD-L1 treatment combine to enhance anti-tumor responses in vivo. **A** Tumor sections were stained for PD-L1 in paraffin-embedded samples. AEC-positive area [%] is presented as individual values in a box-and-whisker plot. Scale bar: 50 µm. Statistical analysis: unpaired t test. Sample sizes: WT = 11, KO = 13. **B** CD274 (protein PD-L1) mRNA expression was analyzed by qPCR in macrophages sorted from tumors. Relative expression [%] is shown as individual values in a box-and-whisker plot. Statistical analysis: unpaired t test (*N* = 5). **C** RAW264.7 and B16-F10 cells were co-cultured and treated for 24 h in starved medium with DMSO, 1 µM MK-2206, DMSO + 50 U/mL IFN-γ, or MK-2206 + IFN-γ. PD-L1–positive B16-F10 cells [%] were quantified by flow cytometry. Cell types were distinguished using CD146, CD45.2, CD11b, and F4/80 markers. Data are presented as mean with SD. Statistical analysis: one-way ANOVA (*N* = 3). **D** B16-F10 cells were injected into C57BL/6 WT mice and treated with vehicle control, MK-2206, anti–PD-L1, or a combination of MK-2206 + anti–PD-L1. Tumor volume (mm³) is shown as mean ± SEM (*N* = 3). **E–G** Tumor sections were stained for (**E**) F4/80, (**F**) PD-L1, and (**G**) STAT1 pY701, STAT1 pS727, and MHC class II. AEC-positive clusters and area [%] are shown as individual values in box-and-whisker plots. Statistical analysis: one-way ANOVA (*N* = 6). *P*-values: * ≤ 0.05, ** ≤ 0.01, **** ≤ 0.0001.

Another important factor in the inflammatory response is the suppression of immune cells. When PD-L1 is expressed on tumor cells or antigen-presenting cells such as macrophages, it binds to its receptor PD-1 on lymphocytes, leading to suppression of these cells [18, 19]. Therefore, we stained tumor tissue for PD-L1 and, importantly, found its expression to be significantly decreased in the KO tumors (Fig. 4A). These results were verified on mRNA level, showing a significant decrease of PD-L1 expression in KO macrophages compared to WT macrophages (Fig. 4B). When co-culturing RAW264.7 with B16-F10 cells, MK-2206 treatment also decreased PD-L1 expression compared to IFN-γ treatment alone on B16-F10 cells (Fig. 4C, Fig. S6A). Hence, we treated B16-F10 tumor bearing mice with MK-2206 alone or in combination with anti-PD-L1 and monitored tumor growth. While the single treatments showed a similar decrease in tumor size compared to the control, the combination treatment showed the smallest tumors, indicating a potentiated effect of MK-2206 and anti-PD-L1 (Fig. 4D). This effect on the tumor size matches the macrophage contents in the tumors, with the smallest tumors having the highest macrophage numbers (Fig. 4E). MK-2206 treatment alone, as well as in combination with anti-PD-L1, decreased PD-L1 expression in the tumors (Fig. 4F). When checking the activating phosphorylation sites of STAT1 and MHCII, the highest expressions can be seen in the combination treatment (Fig. 4G). These results indicate that the combination of currently used anti-PD-L1 treatments with MK-2206 might results in an advantage over the single treatment.

### Loss of IGF-1 during inflammatory response

**Fig. 5:**
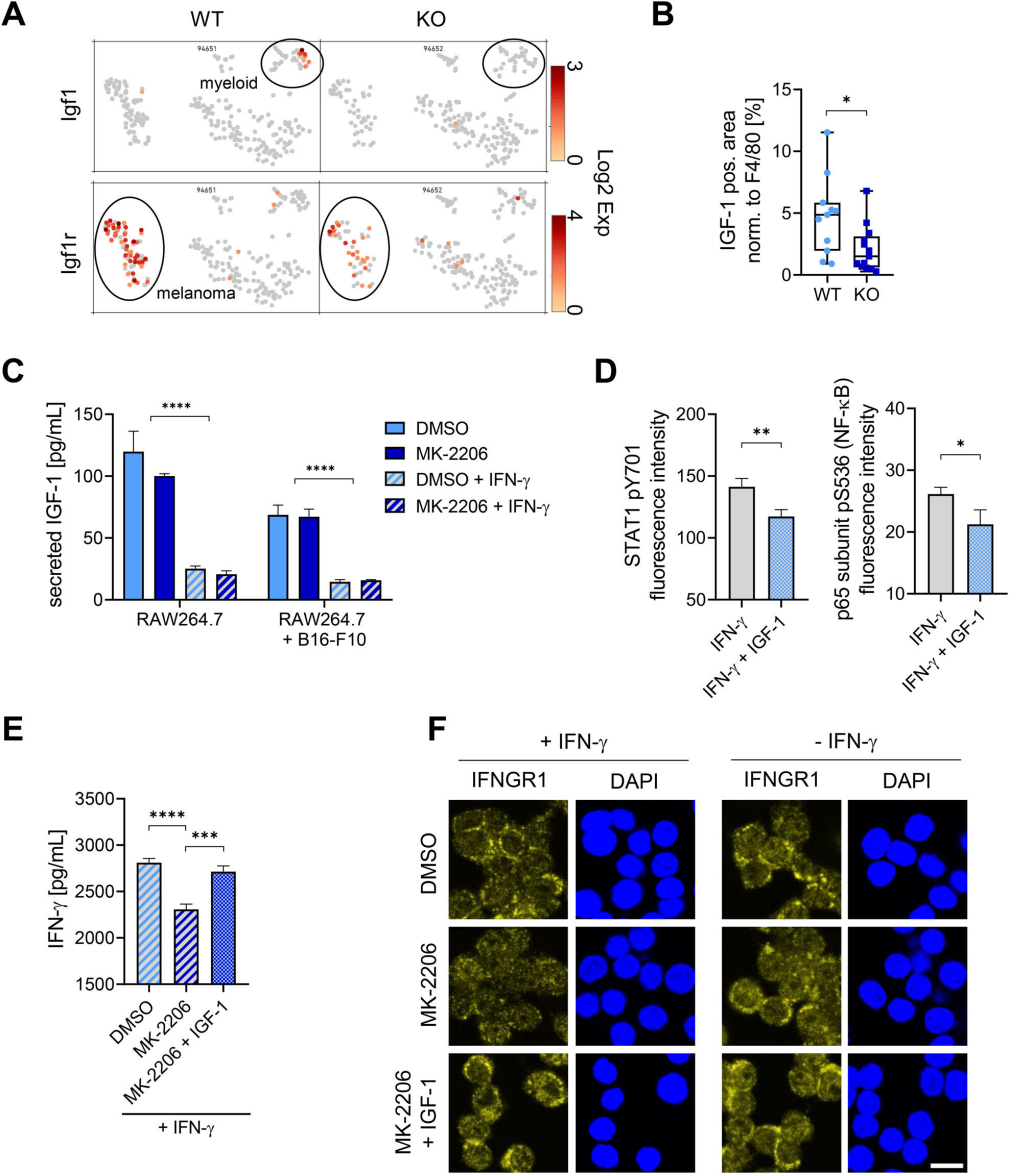
Loss of IGF-1 as a potential mediator of the anti-tumor effect. **A** Single-cell gene expression analysis of tumors showing Igf1 and Igf1r expression levels (Log₂ expression) across individual cells. Clusters corresponding to melanoma cells (bottom left) and myeloid cells (top right) are circled. **B** Tumor sections were stained for IGF-1 in paraffin-embedded samples. AEC-positive area was normalized to F4/80 staining [%] and is presented as individual values in a box-and-whisker plot. Statistical analysis: unpaired t-test. Sample sizes: WT = 11, KO = 13. **C** RAW264.7 cells alone or co-cultured with B16-F10 cells were treated for 72 h with DMSO, 1 µM MK-2206, DMSO + 50 U/mL IFN-γ, or MK-2206 + IFN-γ. IGF-1 levels in the supernatant [pg/mL] were measured by ELISA. Data are shown as mean with SD. Statistical analysis: one-way ANOVA (*N* = 3). **D** B16-F10 cells were treated with 50 U/mL IFN-γ or IFN-γ + 200 ng/mL IGF-1 for 1 h. Phosphorylated STAT1 (pY701) and NF-κB p65 (pS536) were assessed using in-cell western assay. Results are shown as mean fluorescence intensity with SD. Statistical analysis: unpaired t test (*N* = 3). **E** RAW264.7 cells were treated for 24 h with DMSO, 1 µM MK-2206, or MK-2206 + 200 ng/mL IGF-1 in the presence of 50 U/mL IFN-γ. IFN-γ levels in the supernatant [pg/mL] were quantified by ELISA. Data are shown as mean with SD. Statistical analysis: one-way ANOVA (*N* = 3). **F** RAW264.7 cells were treated for 24 h with DMSO, 1 µM MK-2206, or MK-2206 + 200 ng/mL IGF-1, with or without a 10 min treatment with 50 U/mL IFN-γ. Representative immunofluorescence images are shown. Scale bar: 10 µm. *P*-values: * ≤ 0.05, ** ≤ 0.01, *** ≤ 0.001, **** ≤ 0.0001.

We screened our single cell gene expression data for secreted factors and identified loss of IGF-1 expression specifically in Rictor KO myeloid cells. IGF-1 is an anti-inflammatory molecule important for proliferation and development. It is linked to the mTOR pathway and the development and progression of cancer as well as therapy resistance [20]. In the tumors we analyzed, Igf1 was exclusively expressed in WT myeloid cells, but not in KO myeloid cells, while its receptor, Igf1r, was highly expressed in WT melanoma cells compared to KO melanoma cells (Fig. 5A, Fig. S7A). The decrease of IGF-1 was verified on protein level by staining tumor sections (Fig. 5B, Fig. S7B). When treating RAW264.7 macrophages alone or in co-culture with B16-F10 melanoma cells, we found that IFN-γ significantly decreased the amount of secreted IGF-1 (Fig. 5C). These results were verified in human macrophages (Fig. S7C). Therefore, we checked if the effect on the inflammatory pathways could be rescued by IGF-1. Since only melanoma cells express the receptor for IGF-1, B16-F10 cells were treated with IFN-γ alone or in combination with IGF-1. IGF-1 was able to significantly decrease the activating phosphorylations of STAT1 and the p65 subunit of NF-κB (Fig. 5D). Interestingly, we found less IFN-γ in the supernatant of MK-2206 treated RAW264.7 cells (Fig. 5E). This effect could also be rescued by addition of IGF-1. We hypothesize that MK-2206 treatment leads to an increased uptake of IFN-γ, explaining the decreased levels in the supernatant. After binding of IFN-γ, its receptor IFNGR1 gets internalized and inactivated [21, 22]. We therefore checked the presence of the receptor and found it decreased after MK-2206 treatment (Fig. 5F), indicating that in the presence of IGF-1, macrophages respond less to the presence of IFN-γ and therefore show a reduced inflammatory response. These results suggest that IGF-1 works as a crucial suppressor in the inflammatory response and is downregulated by IFN-γ.

## Discussion

**Fig. 6:**
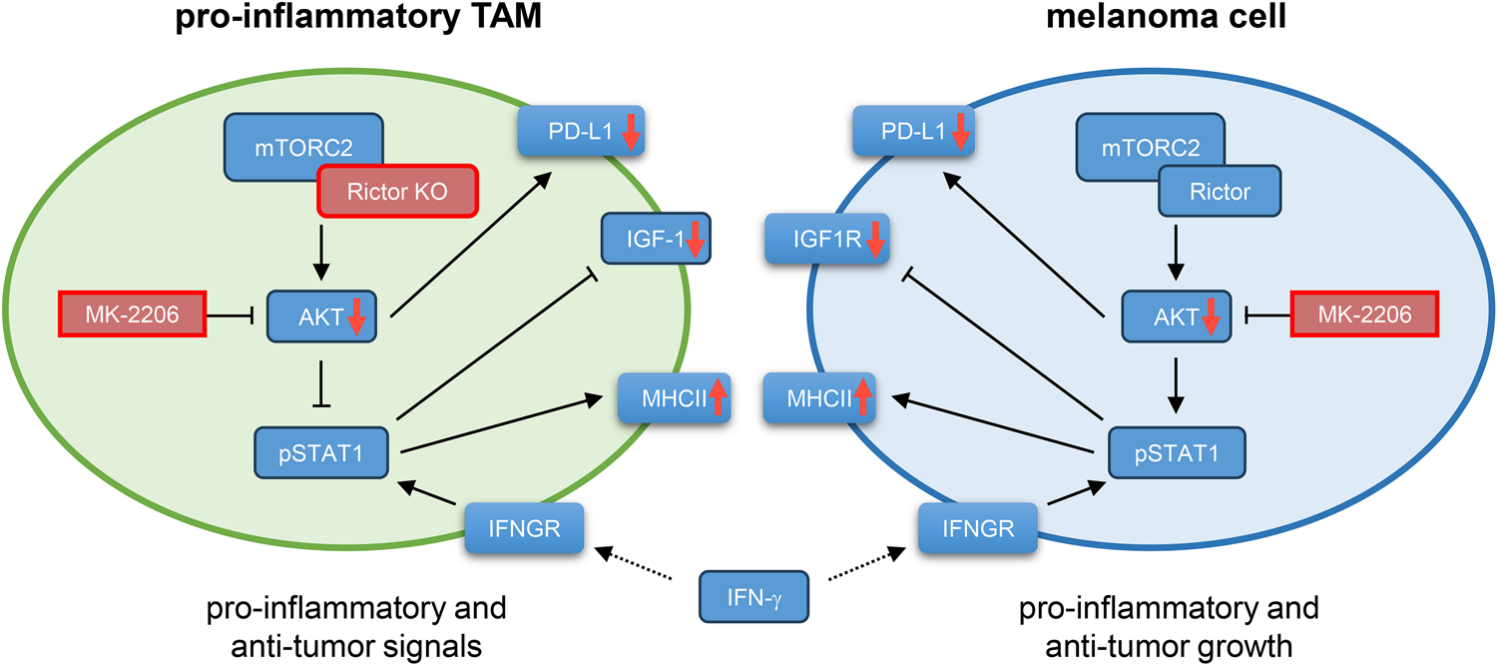
Schematic summary of regulatory changes in tumor-associated macrophages (TAMs) and melanoma cells following myeloid-specific Rictor knockout (KO) and MK-2206 treatment. Red boxes and arrows indicate changes observed in KO or treated cells compared with WT or untreated controls. Arrows represent activating interactions, whereas blunt-ended lines indicate inhibitory effects. Receptors are shown without outlines.

Here, we showed that mTORC2 inhibition decreases the tumor burden in two mouse models as well as in a human skin organoid model by enhancing the inflammatory response. A simplified scheme of regulations seen in TAMs and melanoma cells are summarized in Fig. 6.

The mTOR signaling pathway is frequently upregulated across various cancer types, promoting uncontrolled tumor cell growth and proliferation, and recently, the importance of mTORC2 for cancer cell metabolic reprogramming, survival, and growth has been acknowledged [12–14]. In melanoma, upregulation is commonly driven by mutations in PTEN and PI3K, which in turn activate AKT, a key downstream effector of mTORC2 [23, 24]. The AKT-specific inhibitor MK-2206 has progressed to phase II clinical trials for advanced breast cancer [16] and phase I trials for multiple solid tumors, including melanoma, demonstrating favorable tolerability but variable therapeutic efficacy [25]. In our study, systemic administration of MK-2206 resulted in reduced tumor growth in both a murine melanoma model and a human skin organoid system. Notably, MK-2206 treatment was associated with an inflammatory response, characterized by upregulation of the IFN-γ/STAT1 signaling axis and increased expression of MHC class II molecules. Interestingly, these effects were not observed in monocultures of melanoma cells treated with MK-2206 but were evident in co-cultures with macrophages, suggesting a key role for the tumor microenvironment. This macrophage dependency was further supported by our organoid experiments, where reduced tumor formation was observed only in the presence of macrophages, irrespective of MK-2206 treatment.

Beyond its role in tumor cells, the mTOR signaling pathway balances the differentiation and functional state of immune cells within the tumor microenvironment. In macrophages, mTORC2 signaling is necessary for M2-like differentiation [14]. In our study, we demonstrated that genetic deletion of Rictor in myeloid cells induces a shift in TAMs toward a pro-inflammatory phenotype. This was accompanied by increased phosphorylation of STAT1 at two key activation sites, Tyr701 and Ser727. Canonically, IFN-γ signaling induces STAT1 phosphorylation at Tyr701, promoting dimerization and nuclear translocation. For full transcriptional activity, STAT1 also requires phosphorylation at Ser727, which can be triggered by cellular stress via p38 MAP kinase signaling [26, 27]. These findings suggest that mTORC2 inhibition in macrophages promotes a pro-inflammatory environment, potentially enhancing anti-tumor immunity. Interestingly, autophagy is regulated by the mTOR pathway and plays a dual role in melanoma progression. In early disease stages, it acts as a tumor suppressor by preserving cellular homeostasis, whereas in later stages it promotes tumor growth and survival [28]. Moreover, autophagy in TAMs has been linked to polarization toward an anti-inflammatory phenotype [29]. Notably, studies on melanoma treatment with MK-2206 in combination with chemotherapy demonstrated that inhibition of autophagy prevented the development of resistance [30].

The immune checkpoint molecule PD-L1 is a well-established downstream target of the IFN-γ/STAT1 signaling axis, with STAT1 activation typically leading to increased PD-L1 expression [31, 32]. However, in our study, we observed enhanced STAT1 signaling accompanied by a reduction in PD-L1 expression. This suggests additional layers of regulation. Indeed, PD-L1 expression is also modulated by mTOR signaling, though previous studies have reported conflicting results regarding the direction of this regulation [33–35]. Our findings clearly demonstrate that mTORC2 inhibition in macrophages leads to reduced PD-L1 expression on both macrophages and melanoma cells. Moreover, MK-2206 treatment enhanced the efficacy of anti-PD-L1 therapy in vivo. Given that anti-PD-L1 therapies represent a cornerstone of immunotherapy for advanced melanoma, yet still fail in a significant proportion of patients [36], our data support a combination strategy that may improve treatment responses.

We identified a robust regulation of IGF-1, a macrophage-derived cytokine known for its anti-inflammatory properties and its association with resistance to immune checkpoint blockade [37–39]. High IGF-1 receptor expression in melanoma correlates with increased tumor progression [40]. In our study, we observed IFN-γ–dependent downregulation of IGF-1 in macrophages, and notably, the addition of exogenous IGF-1 reversed the pro-inflammatory effects induced by MK-2206 and IFN-γ. Mechanistically, AKT inhibition reduced membrane localization of IFNGR1, an effect that was rescued by IGF-1 treatment. This aligns with prior findings showing that IGF-1 regulates IFNGR2 expression [41], supporting our conclusion that IGF-1 modulates interferon receptor dynamics. Together, our data suggest that loss of IGF-1 is a hallmark of a pro-inflammatory macrophage phenotype and may potentiate IFN-γ signaling. Targeting the IGF-1/AKT axis could thus represent a promising approach to enhance anti-tumor immune responses in melanoma.

Based on our findings, we propose that combining anti-PD-L1 therapy with either AKT inhibition or IGF-1 pathway inhibition represents a promising therapeutic strategy for melanoma. Notably, recent studies have demonstrated that type I interferons, which also activate the STAT1 pathway, can enhance the efficacy of anti-PD-1 therapies in melanoma [42, 43]. While MK-2206 is currently undergoing clinical evaluation for various cancers [16, 25], the AKT inhibitor capivasertib, developed by AstraZeneca, received EMA approval for the treatment of breast cancer in 2024. A study with MK-2206 alone, in patients with incurable adenoid cystic carcinoma, showed no clinical response, but stable disease in 13 out of 14 patients [44]. Additionally, anti-IGF-1R antibodies are being assessed in phase I clinical trials [45–47], and may serve as a viable alternative for targeting the IGF-1 pathway. Collectively, our data support the potential of mTORC2 inhibition to improve the outcomes of immunotherapy in advanced melanoma, offering a compelling approach toward more effective and potentially curative treatment regimens.

### Materials and methods

#### Mice strains, cell lines and reagents

Female C57BL/6 Rictor floxed +/− LysMcre or C57BL/6 WT mice were used for grafting of B16-F10 cells intradermally. Tumor volume was calculated using the following equation: (width*width*length)/2. Individual mice were treated with MK-2206 (MedChemExpress, NJ, USA) and anti-PD-L1 (Bio X Cell, NH, USA). All animal procedures were approved by the “Animal Care and Use Committee” of the Medical University of Vienna. All methods were carried out in accordance with the approved guidelines of the Animal Care Committee. Experiment was single blinded and no randomization of groups was performed. The mouse macrophage cell line RAW264.7 and mouse melanoma cell line B16-F10 were purchased from ATCC. Cells were cultured in DMEM medium (Gibco, MA, USA) containing 10% FCS (Gibco), 1% penicillin-streptomycin (Sigma-Aldrich, MO, USA), and 2 mM L-glutamine (Sigma-Aldrich). The human melanoma cell line MCM1DLN was generated by Swoboda et al. and cultured in MIM medium containing 2% FCS [48]. Human pluripotent stem cells H9 and WA19 were commercially obtained (WiCell, WI, USA). Cells were tested for mycoplasma contamination regularly. Cells were treated with DMSO (Sigma-Aldrich), MK-2206 (MedChemExpress), IFN-γ (Peprotech, NJ, USA), and IGF-1 (Peprotech).

#### Immunohistochemistry

Tissues from mice or organoids were fixed in 4% formaldehyde. Fixed tissues were dehydrated in 70% ethanol, 100% ethanol, and isopropanol using the KOS Multifunctional Tissue Processor (Milestone, Valbrembo, Italy). Subsequently tissues were embedded in paraffin blocks and cut into 4 µm sections. Antibody staining was performed as described previously [49]. Primary antibodies were F4/80 (1:800, Invitrogen, MA, USA), STAT1 pY701 (1:800, Cell Signaling, MA, USA), STAT1 pS727 (1:800, Cell Signaling), MHC class II (1:100, Abcam, Cambridge, USA), PD-L1 (1:400, Bioss, MA, USA), IGF-1 (1:800, Bioss) and CD11b (1:200, R&D Systems, MN, USA). Stained slides were imaged on the Olympus BX63 Intelligent Microscope (Tokio, Japan) and analyzed using Adobe Photoshop CC 2014.0.0 and ImageJ 1.54 f.

#### Flow cytometry and single cell gene expression analysis of tumors

Tumors from C57BL/6 Rictor floxed +/− LysMcre mice, after 18 days of growth, were dissociated into single cells using the MACS dissociator (Miltenyi Biotech, Bergisch Gladbach, Germany) and either subjected to chromium single cell feature barcoding or stained for CD45, CD11b, Ly6G, Ly6C, F4/80, CD11c and CD24 and analyzed using the Beckman Coulter CytoFLEX S flow cytometer (CA, USA) and the software CytExpert 2.4.0.28. 10X Genomics pipeline was used for generating single cell gene expression data. Data was visualized and analyzed using the Loupe Browser v6.0.0.

#### Quantitative polymerase chain reaction of tumor derived macrophages

Macrophages were sorted from dissociated tumors by using the marker proteins CD45.2 and F4/80. mRNA was isolated (Promega, Madison, WI, USA), transcribed into cDNA (Promega) and the genes Nos2, Tnf, Arg1 and Fizz1 were quantified using TaqMan assays (Thermo Fisher Scientific, Waltham, MA, USA).

#### Flow cytometry of single and co-cultures

Cells were harvested by scraping, followed by a PBS wash, 15 min live/dead staining using Zombie-NIR (1:2000, Biolegend, CA, USA), 2x wash and 10 min blocking using TruStain FcX (1:100, Biolegend) at 4 °C. Cells were incubated with antibodies for extracellular staining for 40 min at 4 °C. Antibodies were CD45.2, CD11b, F4/80, CD146, MHCII, PD-L1. All of them were purchased from Biolegend and diluted 1:200. Subsequently, cells were fixed, permeabilized and stained with STAT1 pY701 PE (1:400, Invitrogen) for 20 min. For S phase measurement the Click-iT™ EdU Pacific Blue™ Flow Cytometry Assay Kit (Invitrogen) was used according to manufacturer´s instructions. Cells were analyzed at the Beckman Coulter CytoFLEX S flow cytometer with the software CytExpert 2.4.0.28.

#### In-cell western assay, Immunofluorescence

Cells were fixed with 4% formaldehyde, washed with PBS and fixed and permeabilized with MeOH for 4 h at −20 °C. Subsequently, cells were blocked with blocking buffer for 1 h and incubated with primary antibody overnight at 4 °C. Primary antibodies were STAT1 pY701 (1:300, Cell Signaling), STAT1 pS727 (1:400, Cell Signaling), MHCII (1:400, Proteintech, IL, USA). The next day, the plate was incubated with secondary antibody (1:500, anti-rabbit 800 CW, Licor, NE, USA) and Celltag (1:1000, 700 CW, Licor) for normalization for 1 h, followed by drying for 1 h. Fluorescence intensities were measured using the Licor Odyssey CLx Imager Licor and quantified on the software Image Studio V5.2.5. For immunofluorescence, treatment was identical, but the primary antibody was IFNGR1 (1:150, Invitrogen) and secondary antibody was AF555 anti-rabbit (1:500, Biolegend) and DAPI (1:5000). Pictures were taken on the Leica TCS Confocal Microscope (Wetzlar, Germany).

#### ELISA

For measurement of IGF-1, cell supernatant was collected after 72 h of culture and quantified using the mouse IGF-1 ELISA Kit (RayBiotech, GA, USA). For IFN-γ, supernatant was collected after 24 h, and the ELISA MAX Deluxe Set Mouse IFN-γ (Biolegend) was used. Protocol was done according to manufacturer´s instructions.

#### Organoid formation and tumor sphere grafting

Human skin organoids were formed out of H9 and WA19 human embryonic stem cells following the protocol of Lee et al. [50]. At day 66, tumor spheroids were attached to the organoids by cultivating them together in a low attachment U bottom plate. Tumor spheroids were generated from 2000 GFP positive MCM1DLN cells alone or 2000 GFP positive MCM1DLN cells + with 2000 human peripheral blood-derived M0 macrophages (STEMCELL Technologies, Vancouver, Canada). After successful attachment, these tumor organoids were treated with IFN-γ or IFN-γ + MK-2206. Half of the culture media was renewed every second day. Pictures of the growing spheroids were taken daily at the Olympus APX100 APEXVIEW microscope. MCM1DLN cells were labelled with GFP using lentiviral infection according to protocol from Schörghofer et al. [51].

#### Statistical analysis

All statistical analysis were done with GraphPad Prism 8.0.1. Results are shown as bar graphs plotting the mean +/− SD, as box & whiskers plots showing all data points or as superimposed symbols plotting the mean +/− SEM. Calculations were done using two-tailed unpaired t tests, one-way ANOVA with Tukey´s multiple comparisons or a mixed-effects model with Geisser-Greenhouse correction and Tukey´s or Sidak´s multiple comparisons. All calculations used a 95% confidence interval. Detailed information per graph can be found in the figure legends.

### Supplementary materials

Supplementary material associated with this article can be found in the online version. During the preparation of this work the authors used ChatGPT in order to improve wording of sentences. After using this tool, the authors reviewed and edited the content as needed and take full responsibility for the content of the publication.

## Supplementary information

Supplementary Figures

Uncropped Western Blots

## Data Availability

The transcriptomic data used in this publication have been deposited in NCBI’s Gene Expression Omnibus (GSE300928). Other data and materials in this paper are available upon reasonable request.
