# Supplementary material for: mTORC2 inhibition reduces tumor burden via STAT1 activation and enhanced response to anti–PD-L1 therapy
Source: Cell Death Dis. 2025 Dec 22;16(1):922. doi: 10.1038/s41419-025-08367-5 (PMC12749672; doi:10.1038/s41419-025-08367-5)
Supplement: Supplementary file 1 — Supplementary Figures [file 41419_2025_8367_MOESM1_ESM.pdf]

Suppl Fig1

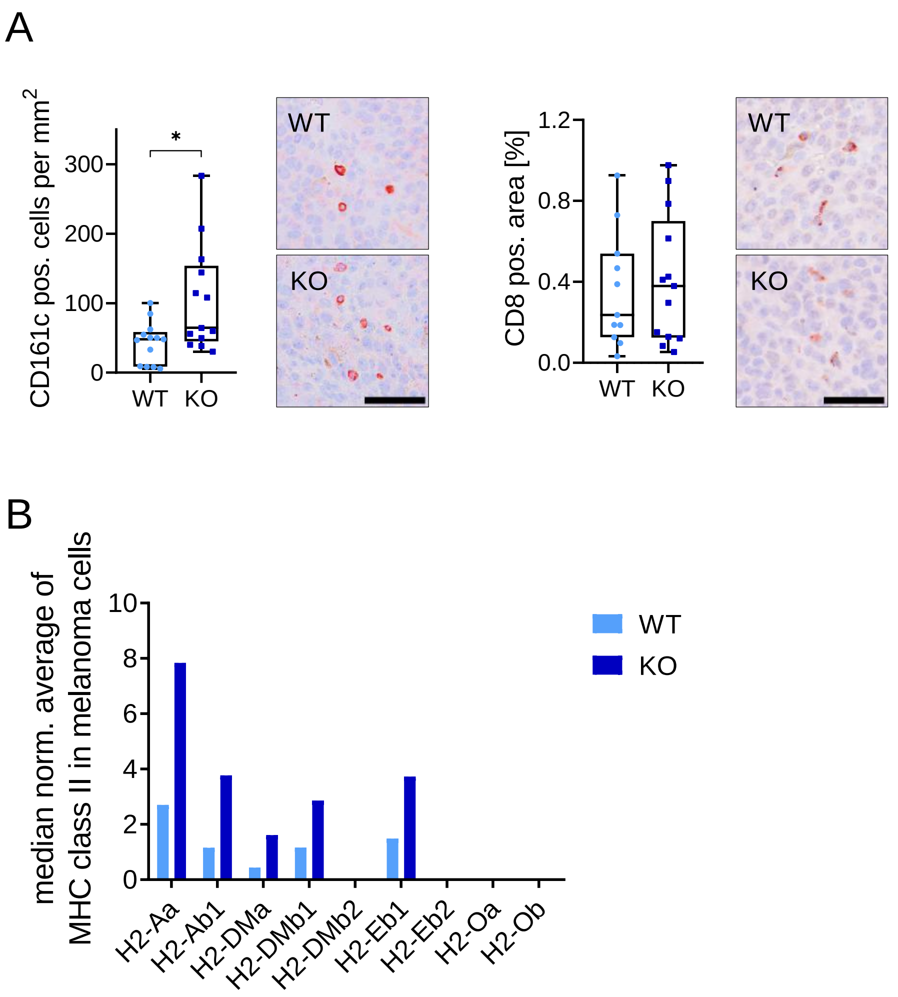

Fig. S1: (A) NK cells and CD8+ T cells in paraffin-embedded tumor sections were stained using the markers CD161c and CD8. Positive cells per mm^2 and AEC-positive area [%] is displayed as individual values in a box-and-whisker plot. Scale bars: 50 µm. Statistical analysis: unpaired t test. Sample sizes: WT = 11, KO = 13. (B) Single cell gene expression analysis of tumors. Median normalized average of isoforms of MHC class II in melanoma cells are shown.

Suppl Fig2

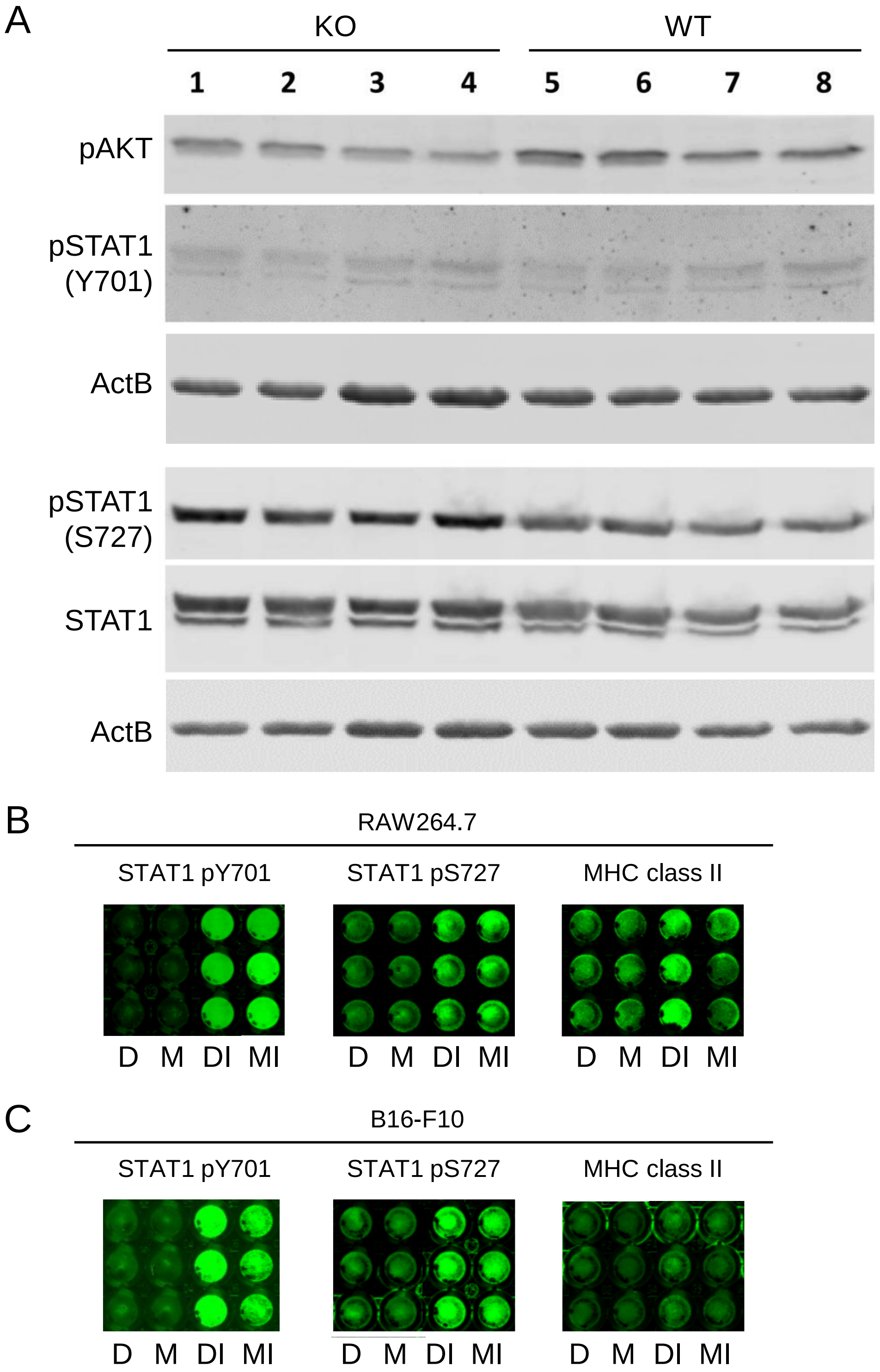

Fig. S2: (A) Western blot from B16-F10-derived tumor tissue grown for 18 days in myleoid specific Rictor KO (sample 1-4) and WT (sample 5-8) C57BL/6 mice. Membranes were stained for AKT phosphorylation at S473, STAT1 phosphorylation at Y701, STAT1 phosphorylation at S727 and total STAT1. Beta Actin is shown as loading control. Uncut membranes can be seen in Fig. S8A. (B, C) Representative antibody stain of STAT1 pY701, STAT1 pS727 and MHC class II of (B) RAW264.7 and (C) B16-F10 cells treated with DMSO (=D), MK-2206 (=M), DMSO + IFN-γ (=DI) or MK-2206 + IFN-γ (=MI) for 24 h.

Suppl Fig3

A

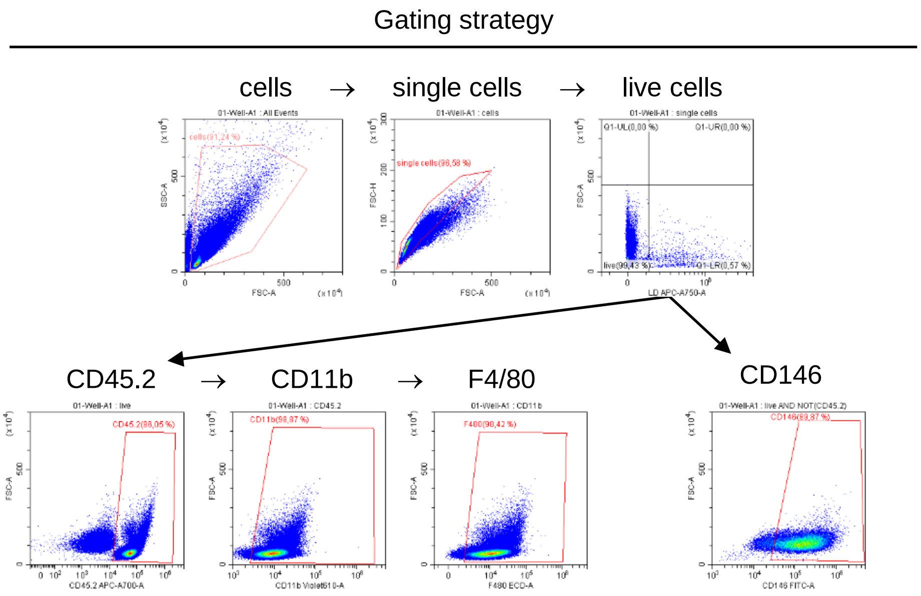

Fig. S3: (A) Representative plots of the gating strategy used for FC experiments. Cells were gated to single and live cells, live cells were further gated using the cell markers CD45.2, CD11b and F4/80 for macrophages or CD146 for melanoma cells.

Suppl Fig4

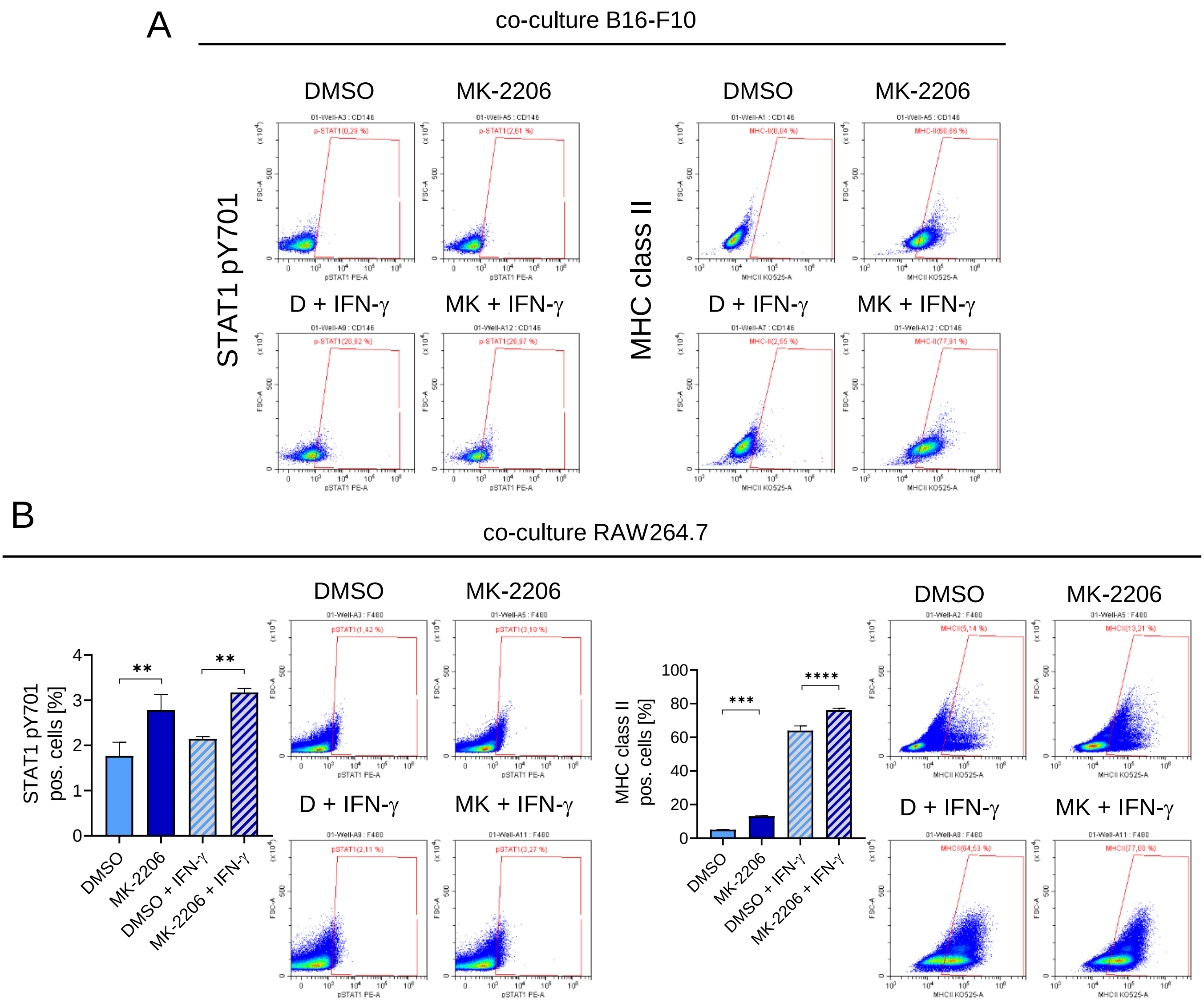

Fig. S4: (A) Representative plots for STAT1 pY701 and MHC class II of B16-F10 cells co-cultured with RAW264.7 cells and treated with DMSO, MK-2206, DMSO + IFN-γ or MK-2206 + IFN- γ for 24 h. B16-F10 cells were distinguished from RAW264.7 cells using the markers CD146, CD45.2, CD11b and F4/80. (B) Same as A, but quantifications and representative plots for RAW264.7 cells are shown. Results are shown as mean with SD. One-way ANOVA was used for statistical analysis (N = 3). P values: ** ≤ 0.01, *** ≤ 0.001, **** ≤ 0.0001.

Suppl Fig5

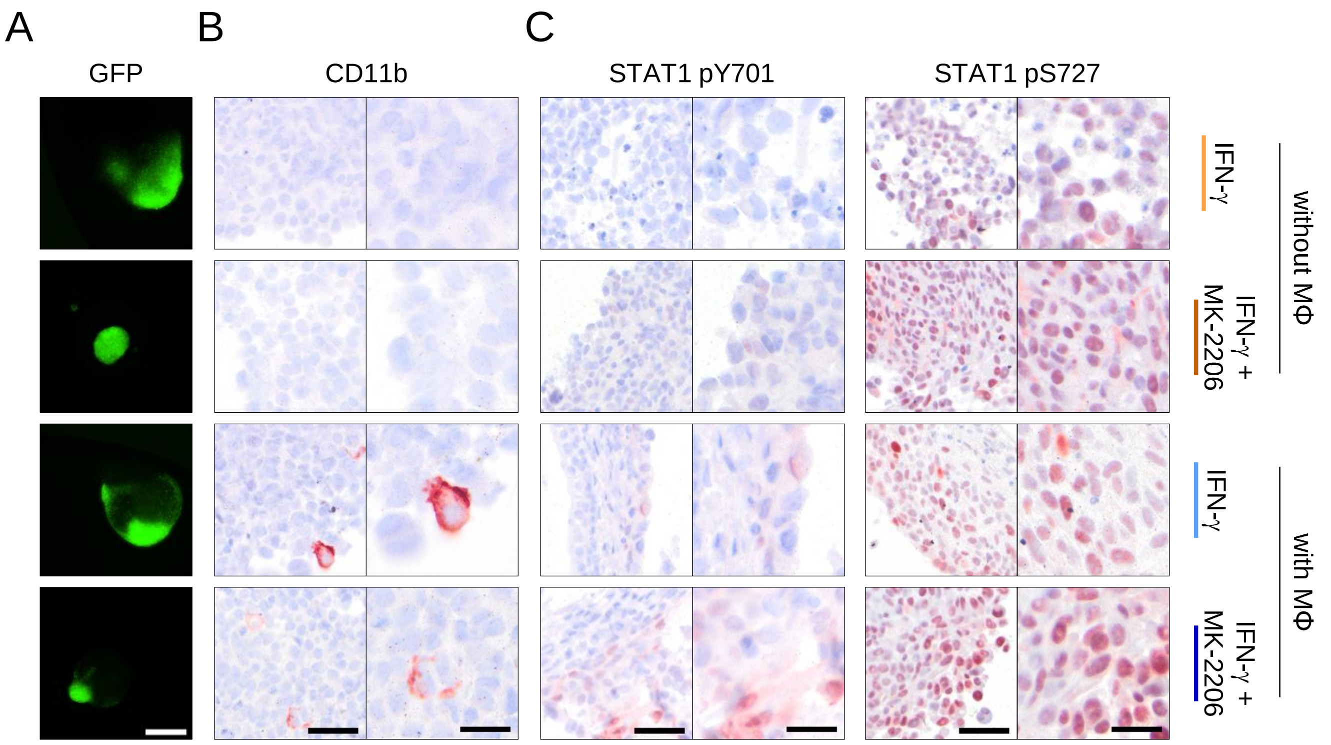

Fig. S5: (A) Representative pictures of GFP labeled MCM1DLN tumor spheroids on WA19 organoids on day 14. Scale bar = 1 mm. (B, C) Representative pictures of (B) CD11b, (C) STAT1 pY701 and STAT1 pS727 stainings of the paraffin-embedded tumor spheroid on organoids. Scale bars: left = 50 µm, right = 25 µm.

Suppl Fig6

A

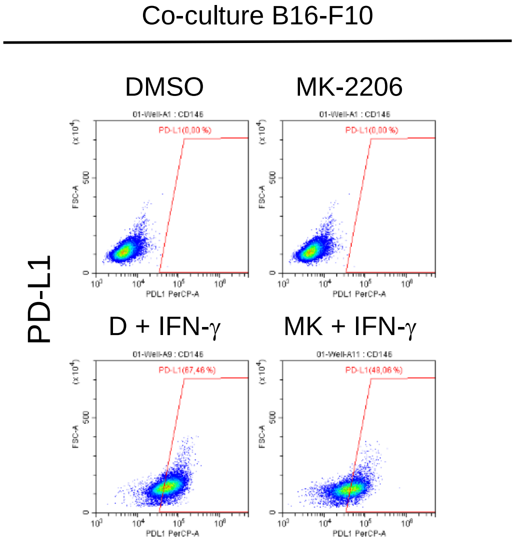

Fig. S6: (A) Representative plots for PD-L1 of B16-F10 cells co-cultured with RAW264.7 cells and treated with DMSO, MK-2206, DMSO + IFN-γ or MK-2206 + IFN- γ for 24 h. B16-F10 cells were distinguished from RAW264.7 cells using the markers CD146, CD45.2, CD11b and F4/80.

Suppl Fig7

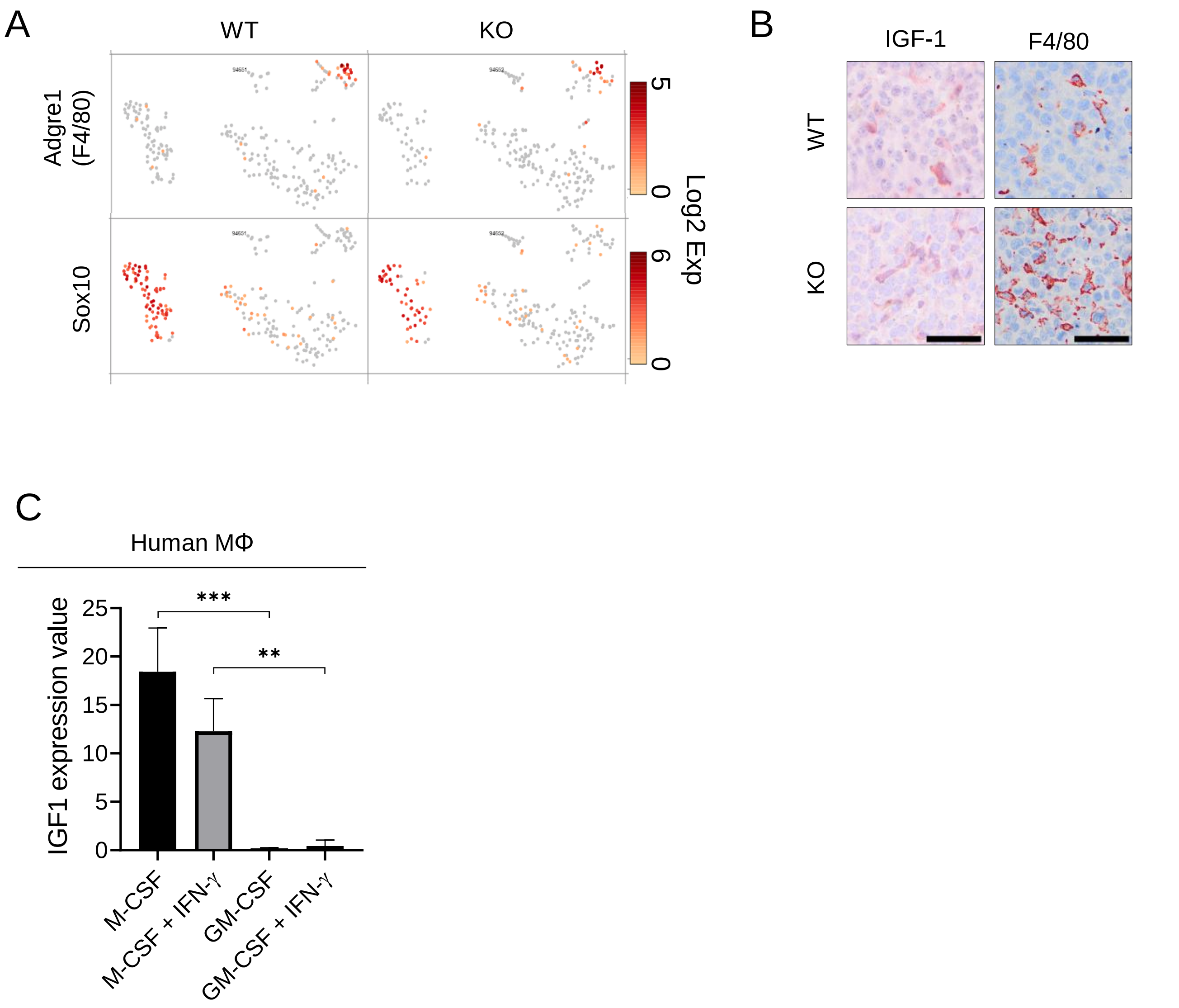

Fig. S7: (A) Single cell gene expression of F4/80 and Sox10 [Log2 Exp] used to identify myeloid and melanoma cells. (B) Representative pictures of paraffin-embedded tumor sections stained for IGF-1 and F4/80. Scale bars = 50 µm. (C) IGF1 expression values of M-CSF, GM-CSF and IFN-γ treated human macrophages from the GSE130567 dataset. Results are shown as mean with SD. One-way ANOVA was used for statistical analysis (N = 3). P values: ** ≤ 0.01, *** ≤ 0.001.
