# Supplementary material for: mTORC2 inhibition reduces tumor burden via STAT1 activation and enhanced response to anti–PD-L1 therapy
Source: Cell Death Dis. 2025 Dec 22;16(1):922. doi: 10.1038/s41419-025-08367-5 (PMC12749672; doi:10.1038/s41419-025-08367-5)
Supplement: Supplementary file 2 — Uncropped Western Blots [file 41419_2025_8367_MOESM2_ESM.pdf]

Suppl Fig2

A

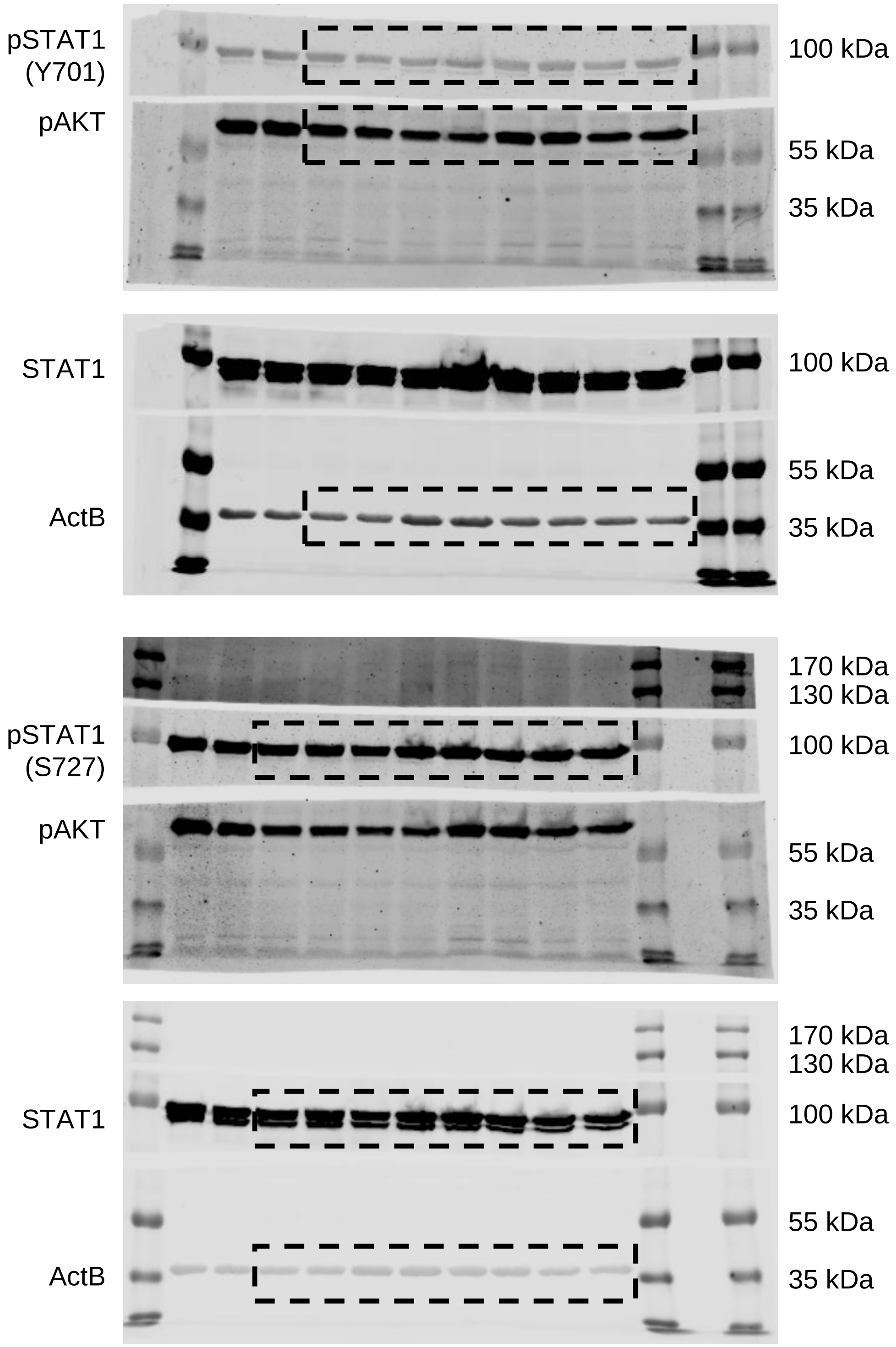

Fig. S2: (A) Uncut western blot seen in Fig. S2A. Samples are framed. Membranes are shown at 680 nm and 800 nm.
